# Supplementary material for: Evaluation of FluSight influenza forecasting in the 2021–22 and 2022–23 seasons with a new target laboratory-confirmed influenza hospitalizations
Source: Nat Commun. 2024 Jul 26;15:6289. doi: 10.1038/s41467-024-50601-9 (PMC11282251; doi:10.1038/s41467-024-50601-9)
Supplement: Supplementary file 1 — Supplmentary Information [file 41467_2024_50601_MOESM1_ESM.pdf]

Evaluation of FluSight influenza forecasting in the 2021-22 and 2022-23 seasons with a new target laboratory-confirmed influenza hospitalizations. Mathis et al.

Table of Contents

Supplementary Methods

Supplementary Table 1: Model information for models meeting primary inclusion criteria

Supplementary Table 2: Model information for models that did not meet inclusion criteria

Supplementary Figure 1: Distribution of relative WIS across subnational jurisdictions

Supplementary Analysis 1. Backfill/Revision of hospital admission data

Supplementary Analysis 2: Performance of log-transformed forecasts

Supplementary Analysis 3. Performance of national forecasts

Supplementary References

Supplementary Methods

We used the following version of relative Weighted Interval Score (WIS) as a main metric of evaluation[1].

$$IS_{\alpha}(F,y) = (u-l) + \frac{2}{\alpha} \cdot (l-y) \cdot 1(y<l) + \frac{2}{\alpha} \cdot (y-u) \cdot 1(y>u)$$

where 1(·) is the indicator function, y is the observed value, with uncertainty level α, and l and u are the $\frac{\alpha}{2}$ and $1-\frac{\alpha}{2}$ quantiles of the predictive distribution F, i.e. the lower and upper bound of a single prediction interval. For a set of K prediction intervals and the median m, the score is computed as a weighted sum,

$$WIS = \frac{1}{K+0.5} \cdot \left(w_0 \cdot |y-m| + \sum_{k=1}^{K} w_k \cdot IS_{\alpha}(F,y) \right)$$

where w_k is a weight for every interval. Usually, $w_k = \frac{\alpha_k}{2}$ and $w_0 = 0.5$.

To compute the relative skill θ_i of model i, we take the geometric mean of all mean score ratios that involve model i, i.e.

$$\theta_i = \left(\prod_{m=1}^{M} \theta_{im} \right)^{\frac{1}{M}}$$

where M is the number of models.

Supplementary Table 1: Types of models included in analysis

Model Name	Type	Methods	Additional data used*	Repo_url
**CEID-Walk** (2021-22)	Statistical	Random walk model without drift. The last few observations of a target time series are used to estimate the variance in step size of the random walk.		
**CEPH-Rtrend_fluH** (2022-23)	Statistical	Renewal equation based on a Bayesian estimation of R(t) from hospitalization data.		https://publichealth.indiana.edu/research/faculty-directory/profile.html?user=majelli https://github.com/paulocv/Rtrend-fluH
**CMU-TimeSeries** (2021-22)	TS regression Statistical	A quantile autoregression fit using influenza-related hospitalizations and doctors visits, jointly trained across locations using the most recently available 21 days of data.	HHS field estimated percentage of outpatient doctors visits with confirmed influenza	https://github.com/cmu-delphi/flu-hosp-forecast/
**CMU-TimeSeries** (2022-23)	TS regression Statistical	A quantile autoregression fit using influenza-related hospitalizations and doctors visits, jointly trained across locations using the most recently available 21 days of data.ensembled with another quantile autoregression model with a longer time window and adjustments for data latency.	HHS field estimated percentage of outpatient doctors visits with confirmed influenza	https://github.com/cmu-delphi/flu-hosp-forecast/
**CU-ensemble** (2021-22,2022-23)	Ensemble	An inverse-WIS weighted ensemble of three models – an SEIRS compartmental model with EAKF, an ARIMA model, and a baseline model of random walk with drift.	"Daily climatology of specific humidity for each state compiled from the National Land Data Assimilation System (NLDAS) project-2 data set"	
**GH-Flusight** (2021-22)	Ensemble	National and State-Level model, providing daily forecast predictions of flu patients admitted to the hospital, providing forecasts for next four weeks. Model was an ensemble development		https://github.com/gh-ai-solu/flusight-21-22
**GT-FluFNP** (2021-22,2022-23)	Statistical	Data-driven approach based on probabilistic non-parametric neural Gaussian processes using syndromic, clinical, demographic, and mobility data.	Demographic, Google mobility, Facebook symptomatic data, JHU-CSSE, COVID-Net	https://github.com/AdityaLab/EpiFNP
**IEM_Health-FluProject** (2021-22)	Statistical	State- and national-level SEIR model projections for weekly incident confirmed flu hospitalizations by using MCMC to fit actual hospitalizations observed.		
**JHU_IDD-CovidSP** (2022-23)	Mechanistic	State-level metapopulation SEIR model with inter-state human mobility, explicit modeling of age-structure, vaccination, immunity waning, and Flu types A and B. The disease transmission is changed over time by non-pharmaceutical/behavioral		https://www.flepimop.org/

		interventions and humidity-driven seasonality.		
**LosAlamos_NAU-Cmodel_Flu** (2021-22)	Mechanistic Statistical	Seasonal compartmental model to perform Bayesian analysis on hospitalization data available for each state.		https://github.com/computationalUncertaintyLab/2022Flu_ LucompUncertLab
**LucompUncertLab-TEVA** (2021-22)	Statistical Ensemble	The four VAR models submitted by LU are combined by Thompson sampling with weights assigned at the location level and proportional to the number of times a model has the lowest WIS in that location.	Four component models submitted by Lehigh University	https://github.com/computationalUncertaintyLab/2022Flu_ LucompUncertLab
**LucompUncertLab-VAR2** (2021-22)	Statistical	VAR(2) model that groups state-level hospitalization trajectories by HHS region and is fit with stan.		https://github.com/computationalUncertaintyLab/2022Flu_ LucompUncertLab
**LucompUncertLab-VAR2_plusCOVID** (2021-22)	Statistical	VAR(2) model that groups state-level hospitalization trajectories due to influenza and COVID-19 and is fit with stan.	HHS state-level confirmed COVID-19 hospitalizations aggregated to weeks.	https://github.com/computationalUncertaintyLab/2022Flu_ LucompUncertLab
**LucompUncertLab-VAR2K** (2021-22)	Statistical	VAR model that groups state-level hospitalization trajectories due to influenza and COVID by HHS region, selects the number of lagged weeks between 1 to 5 weeks behind, and is fit with stan.	HHS state-level confirmed COVID-19 hospitalizations aggregated to weeks.	https://github.com/computationalUncertaintyLab/2022Flu_ LucompUncertLab
**LucompUncertLab-VAR2K_plusCOVID** (2021-22)	Statistical	VAR model that groups state-level hospitalization trajectories due to influenza and COVID-19, probabilistically selects the number of lags from 1 to 5 weeks, and is fit with stan.	HHS state-level confirmed COVID-19 hospitalizations aggregated to weeks.	https://github.com/computationalUncertaintyLab/2022Flu_ LucompUncertLab-VAR2
**MIGHTE-Nsemble** (2022-23)	Machine-Learning Ensemble	Statistical Ensemble encompassing a set of network-based models, including Vector Autoregression and Lasso, as well as Exogenous Data models such as ARGO.	Influenza-related Google Searches retrieved from Google Trends.	
**MOBS-GLEAM_FLUH** (2021-22,2022-23)	Mechanistic Network	Metapopulation, age structured SLIR model, including local mobility and domestic airline traffic and school calendar.	HHS state-level confirmed COVID-19 hospitalizations aggregated to weeks.	https://www.gleamproject.org/flu-forecasting
**PSI-DICE** (2021-22,2022-23)	Mechanistic	A stochastic/deterministic, SIRH model that stratifies by disease severity and includes compartments for hospitalizations. Parameter posteriors are inferred using an MCMC procedure.		
**Sgroup-RandomForest** (2021-22,2022-23)	Ensemble	Random Forest ensemble of the predictors generated from the Sgroup-SikJalpha submission, HHS data, and FluSurv-NET data.	FluSurv-NET	https://github.com/maa989/RandomForest-SikJalpha
**Sgroup-SIkJalpha** (2021-22)	Statistical	Discrete heterogeneous rate model where rates are learned using regression with a forgetting factor that weighs recently seen data higher.		https://github.com/scc-usc/ReCOVER-COVID-19
**SigSci-CREG** (2021-22,2022-23)	Statistical	Count regression model.		https://github.com/signaturescience/fiphde

**SigSci-TSENS** (2021-22,2022-23)	TS ensemble	Ensemble of time series models.		https://github.com/signaturescience/fiphde
**UGA_flucast-OKeeffe** (2022-23)	Statistical	A flexible machine learning model that is coupled with a tempering term that functionally acts similar to how susceptible depletion would in a mechanistic modeling framework.		https://github.com/gcgibson/semimech_package
**Umass-trends_ensemble** (2021-22,2022-23)	TS ensemble	Equally weighted ensemble of simple time-series baseline models.		https://github.com/reichlab/flu-hosp-models-2021-2022
**UNC_IDD-InfluPaint** (2022-23)	Statistical	We build a generative denoising diffusion probabilistic model of Influenza dynamics that generates synthetic epidemic trajectories. We condition these trajectories on ground-truth data using an inpainting algorithm to produce our forecasts.		https://github.com/jcblemai/inpainting-idforecasts
**UT_FluCast-Voltaire** (2021-22)	Statistical	Rolling Bayesian taylor approximation on logarithm of cases implemented in stan.	HHS previous day admission influenza confirmed coverage	
**UVAFluX-Ensemble** (2021-22)	TS ensemble	Ensemble of multiple methods such as auto-regressive (AR) models with exogenous variables, ARIMA, Kalman filter and Long short-term memory (LSTM) models		
**UVAFluX-Ensemble** (2022-23)	TS ensemble	Ensemble of multiple methods such as auto-regressive (AR) models with exogenous variables, ARIMA, Kalman filter, Long short-term memory (LSTM) models, an exponential smoothing model, and a segmented trend forecasting model		
**VTSanghani-ExogModel** (2022-23)	AI	Deep learning model that uses various exogenous datasets	Air Quality Index, and HHS confirmed COVID-19 hospitalizations at the state level and aggregated to weeks.	

*Data used in addition to the HHS COVID-19 Reported Patient Impact and Hospital Capacity by State Timeseries – confirmed influenza hospitalization admissions

Supplementary Table 2: Types of models submitted to FluSight but did not meet inclusion criteria.

Model Name	Type	Methods	Additional data used*	Repo_url
**CADPH-FluCAT_Ensemble** (2022-23)	TS Ensemble	This model is an ensemble of several time series forecasting and predictive models targeting influenza hospital admits based on historical clinical lab and public health lab flu surveillance data.	California Department of Health Care Access and Information	https://calcat.covid19.ca.gov/cacovidmodels/
**CEID-Walk** (2022-23)	Statistical	Random walk model without drift. The last few observations of a target time series are used to estimate the variance in step size of the random walk.		
**ISU_NiemiLab-Flu** (2022-23)	Statistical	By state ARIMA		
**JHUAPL-Gecko** (2021-22)	Time Series	SARIMA time series model (1,1,0)x(1,1,0,7) with anomaly detector applied to confirmed hospital admissions since 09/01/2020.		https://gitlab.jhuapl.edu/panagmj1/gecko-sarima
**LosAlamos_NAU-Cmodel_Flu** (2022-23)	Mechanistic Statistical	Seasonal compartmental model to perform Bayesian analysis on hospitalization data available for each state.		https://github.com/computationalUncertaintyLab/2022Flu_LucompUncertLab
**LUcompUncertLab-ensemble_rclp** (2022-23)	Statistical Ensemble	A model that combines component models and recalibrates using a mixture of beta distributions	Models from LU	https://github.com/computationalUncertaintyLab/
**LUcompUncertLab-experthuman** (2022-23)	Human Judgement	A model built by expert judgement predictions.	Expert judgement prob densities	https://github.com/computationalUncertaintyLab/2022Flu_LUcompUncertLab
**LUcompUncertLab-hier_mech_model** (2022-23)	Hierarchical Mechanistic	We fit a SIRHD compartmental model where S0 is shared between seasons	Weekly num of ILI from ILInet	https://github.com/computationalUncertaintyLab/
**LucompUncertLab-humanjudgement** (2021-22, 2022-23)	Human judgement	A model built by interpolating Human judgement predictions and projecting them from six locations to all 54.	Human judgement probability densities from Metaculus	https://github.com/lanl/SeasonalFlu-2022-Predictions
**LUcompUncertLab-HWAR2** (2022-23)	Statistical	We detrend with Holt Winters and then fit the residuals with a Bayesian AR(2) process		https://github.com/computationalUncertaintyLab/
**LUcompUncertLab-KalmanFilter** (2022-23)	Statistical	A model that supposes there is a ltent AR process that generates observations.		https://github.com/computationalUncertaintyLab/
**LUcompUncertLab-stacked_ili** (2022-23)		A model that incorporates ILI data		https://github.com/computationalUncertaintyLab/
**NIH-Flu_ARIMA** (2022-23)	Statistical	Seasonal ARIMA model with exogenous covariates for cumulative vaccination coverage, seasonal vaccine effectiveness, weekly A/H3N2 circulation, and influenza transmissibility.	CDC FluSurv-NET hospitalization rates, syndromic and virologic surveillance data from CDC FluView, vaccination coverage estimates from the National Center for Immunization and Respiratory Diseases, and seasonal vaccine effectiveness estimates from published	https://github.com/midas-network/flu-scenario-modeling-hub/tree/main/data-processed/NIH-Flu_TS

			observational studies and the CDC	
**UGuelph-FluPLUG** (2022-23)	TS Statistical	Applies piecewise linear segmentation to time series of log(incidence) informed also by data on past seasons.		https://sites.google.com/site/mgcojocarumath/networks-and-dynamics-lab
**Umass-ARIMA** (2021-22)	TS regression Statistcal	Autoregressive integrated moving average of time-series model	MA DPH Syndromic Surveillance (SyS) for 2017 to 2021	https://github.com/reichlab/flu-hosp-models-2021-22
**UMass-gbq** (2022-23)	Statistical	Gradient boosting for the median, followed by conformal prediction to get quantiles.		https://github.com/reichlab/covid-hosp-models
**VTSanghani-ExogModel** (2021-22)	AI	Deep learning model that uses various exogenous datasets	Air Quality Index, and HHS confirmed COVID-19 hospitalizations at the state level and aggregated to weeks.	
**VTSanghani-Transformer** (2022-23)	TS Statistical	A model based on the Temporal Fusion Transformer architecture.		https://sanghani.cs.vt.edu/

Supplementary Figure 1: Distribution of relative WIS across models for all subnational forecast jurisdictions. The horizontal black line is at one, where forecast performance is the same as the Flusight-baseline (see main manuscript Figure 3). Boxplot elements are defined as follows: center line, median; box limits, upper and lower quartiles; whiskers, 1.5x interquartile range; points, outliers.

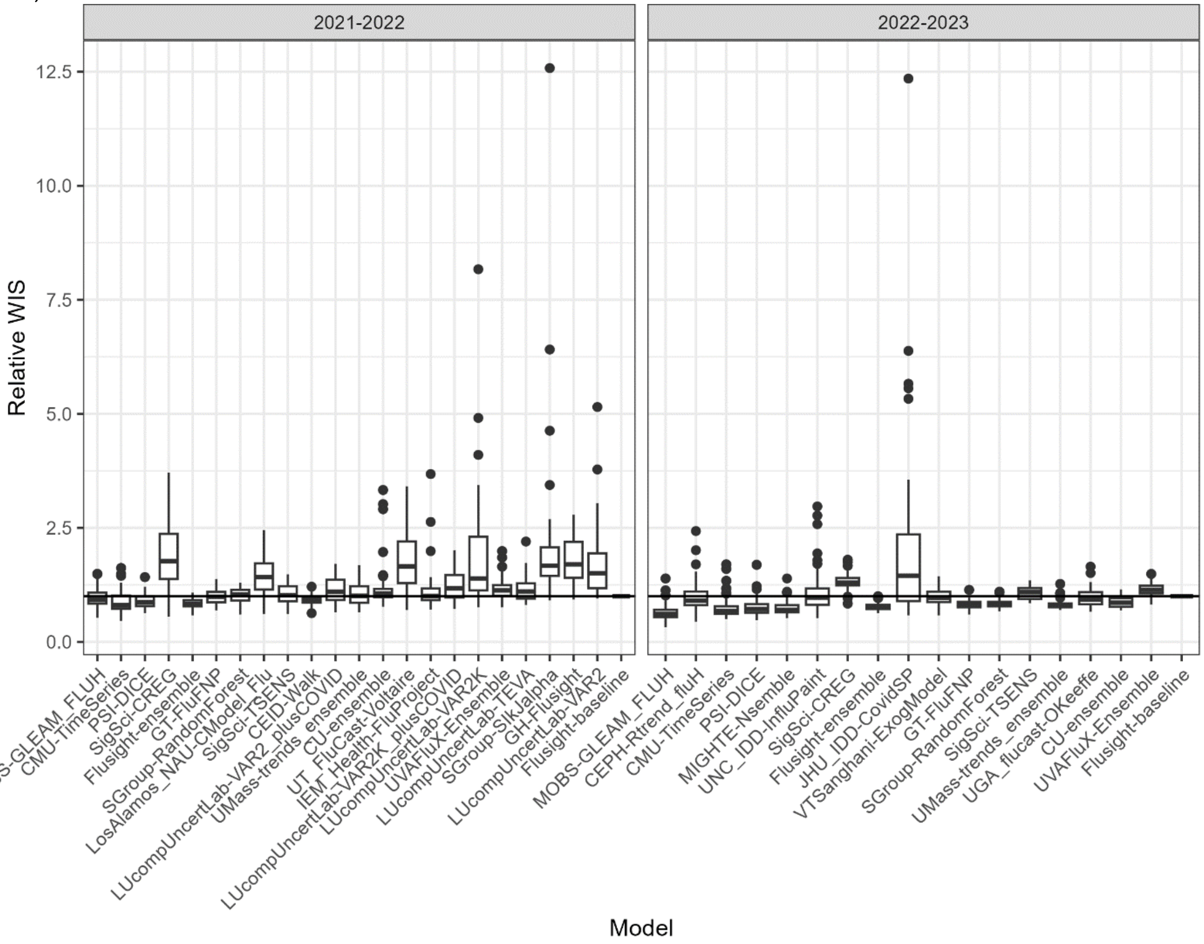

Supplementary Analysis 1: Backfill/Revision of hospital admission data

Methods

Differences between the finalized data used in this evaluation (reported as of September 12, 2022, for the 2021-22 season, and June 13, 2023, for the 2022-23 season) and the originally reported values were analyzed by comparing differences in the weekly incident rate by both the relative change and absolute change. Original reported confirmed influenza hospital admissions were downloaded from the COVID-19 Reported Patient Impact and Hospital Capacity by State Timeseries Archive Repository [2] and aggregated by week for each state and territory. Reported values of weekly hospitalizations were compared each week for the entire U.S. from February 7, 2022, to July 16, 2022, for the 2021-22 season, and October 17, 2022, to June 13, 2023 for the 2022-23 season. This was done to capture updates spanning the start of the mandatory influenza reporting period through the weeks needed to evaluate the last four-week ahead forecasts. For each jurisdiction, we calculated the percent difference and numeric difference between the number of hospitalizations between the initial week of publication and the number of hospitalizations when the data were considered final for these analyses. During the 2022-23 season, it became evident that the frequency and magnitude of backfilled counts was dependent on day of the week. Revisions were more likely to occur on Tuesdays rather than on Mondays. Due to this day of the week revision effect, weekly observed data used in this analysis reflect the days that data were pulled during the season; prior to January 16, data were downloaded each Monday, and beginning January 16, data were downloaded each Tuesday.

Results

To assess potential impacts of changes in reported values on forecast performance, we compared reported incident hospitalizations [3] at the time they would have been used for forecasts to the values used in evaluating forecasts (Supplementary Figures 2–4). Most data updates occurred within one month of when the data were initially published, and 12 out of the 18 weeks that were analyzed did not have updates two weeks after initial publication for 2021-22. Prior to the week ending December 19, most data updates occurred within one month of when the data were initially published. Subsequently, data were updated more frequently over a longer period. For both seasons, across all subnational forecast jurisdictions, a majority (93.96% in 2021-22 and 83.48% in 2022-23) of final values changed by less than ten hospitalizations or did not change compared to corresponding initial reports. In 2021-22, a higher percent of updates had final values lower than initial reports, 37.91%, compared to 30.37% in 2022-23. Conversely, 2022-23 had a higher percent of updates with no change (38.69%) than the 2021-22 season (31.68%). Both seasons had similar percent of updates with final values higher than initial reports (30.94% and 30.4%, for 2022-23 and 2021-22 respectively). For the U.S., the median percent and numeric difference across all forecast weeks were lower in 2021-22, with a median percent difference of -2.1% (range: -4.75% to 0.6%), and the median numeric difference of -39 (range: -157, 19). For 2022-23, the median percent difference across all forecast weeks was 0.46% (range: -7.93% to 6.55%), and the median numeric difference was 14.5 (range: -389, 1,220).

Jurisdiction-specific median percent differences had a larger range in 2021-22 from -14.29% in Rhode Island to 7.14% in Oregon whereas percent differences ranged from -4.53% in Indiana to 4.39% in Illinois for 2022-23. Across states, DC, and Puerto Rico, and for both seasons, the median numeric difference between initial report and final report was 0. Supplementary Figures

2-4 for additional information on the distribution of differences between final reported values used for evaluation and the initial reported values.

Supplementary Figure 2: Data updates analysis: initial epicurve (points available at time of forecast in blue) and final epicurve reported from HHS-Protect as of the final evaluation date of September 12, 2022 (2021-2022, panel a) and June 12, 2023 (2022-2023, panel b) for each season, respectively (in red).

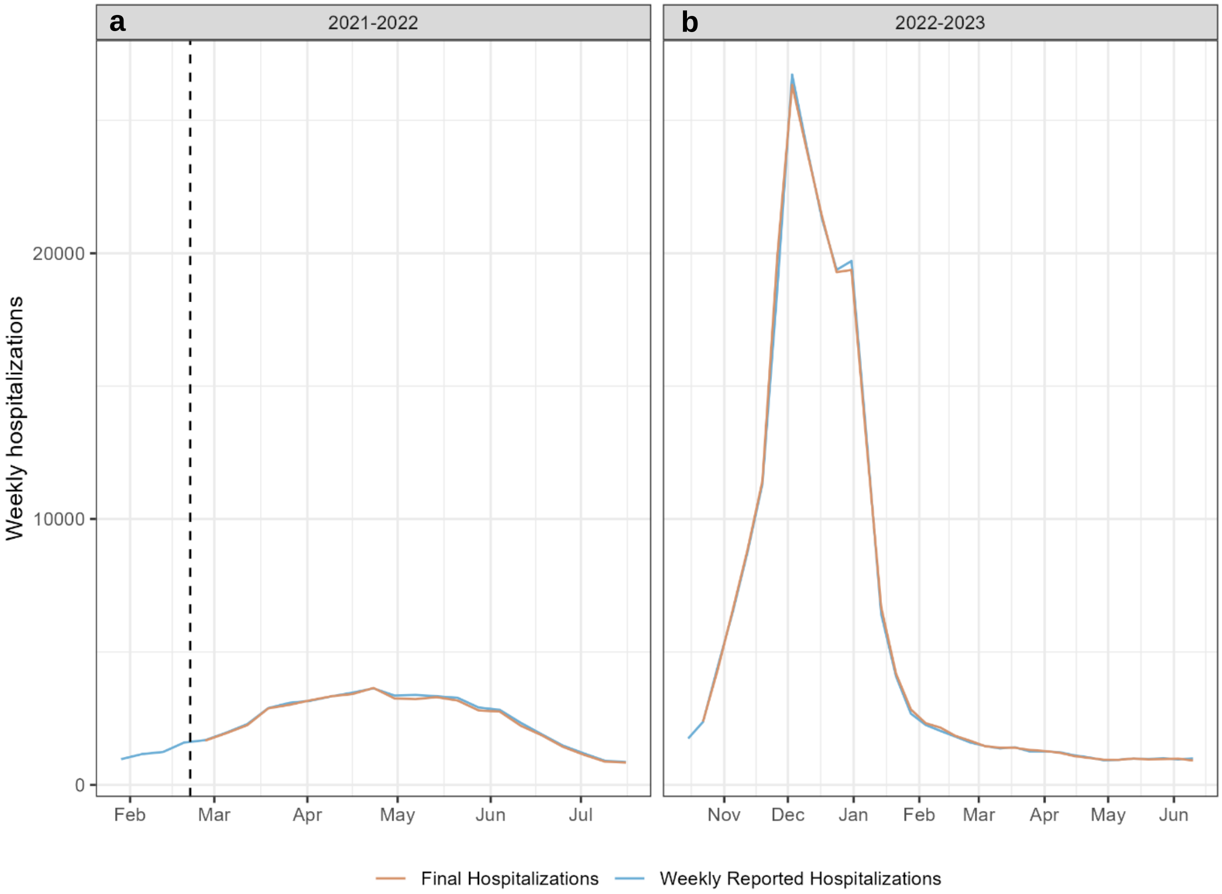

Supplementary Figure 3: state−specific distributions of absolute (panels a and b) and relative changes (panels c and d) in HHS−Protect reported hospitalizations as of the initial report date and the final evaluation date. Boxplot elements are defined as follows: center line, median;box limits, upper and lower quartiles; whiskers, 1.5x interquartile range; points, outliers.

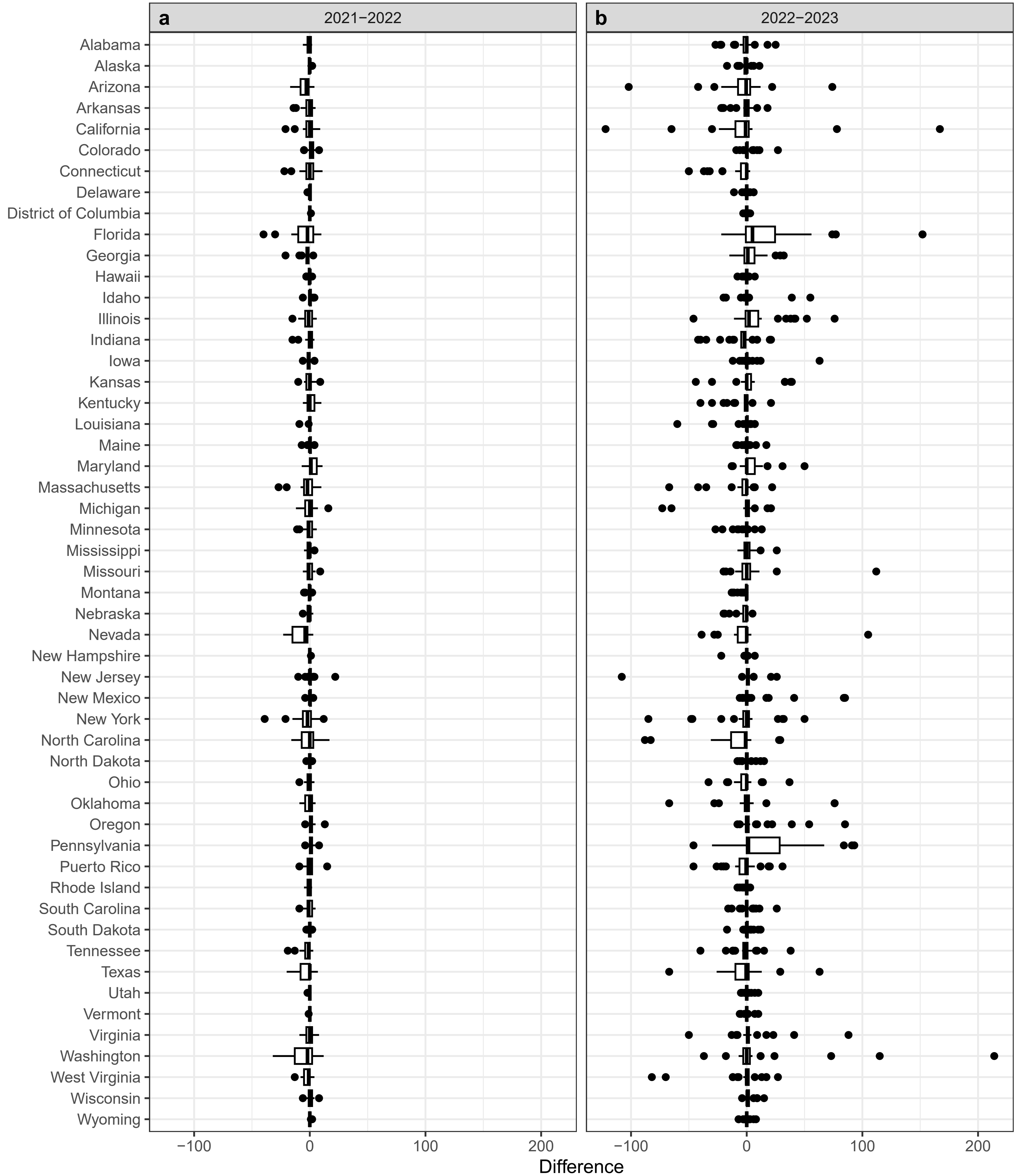

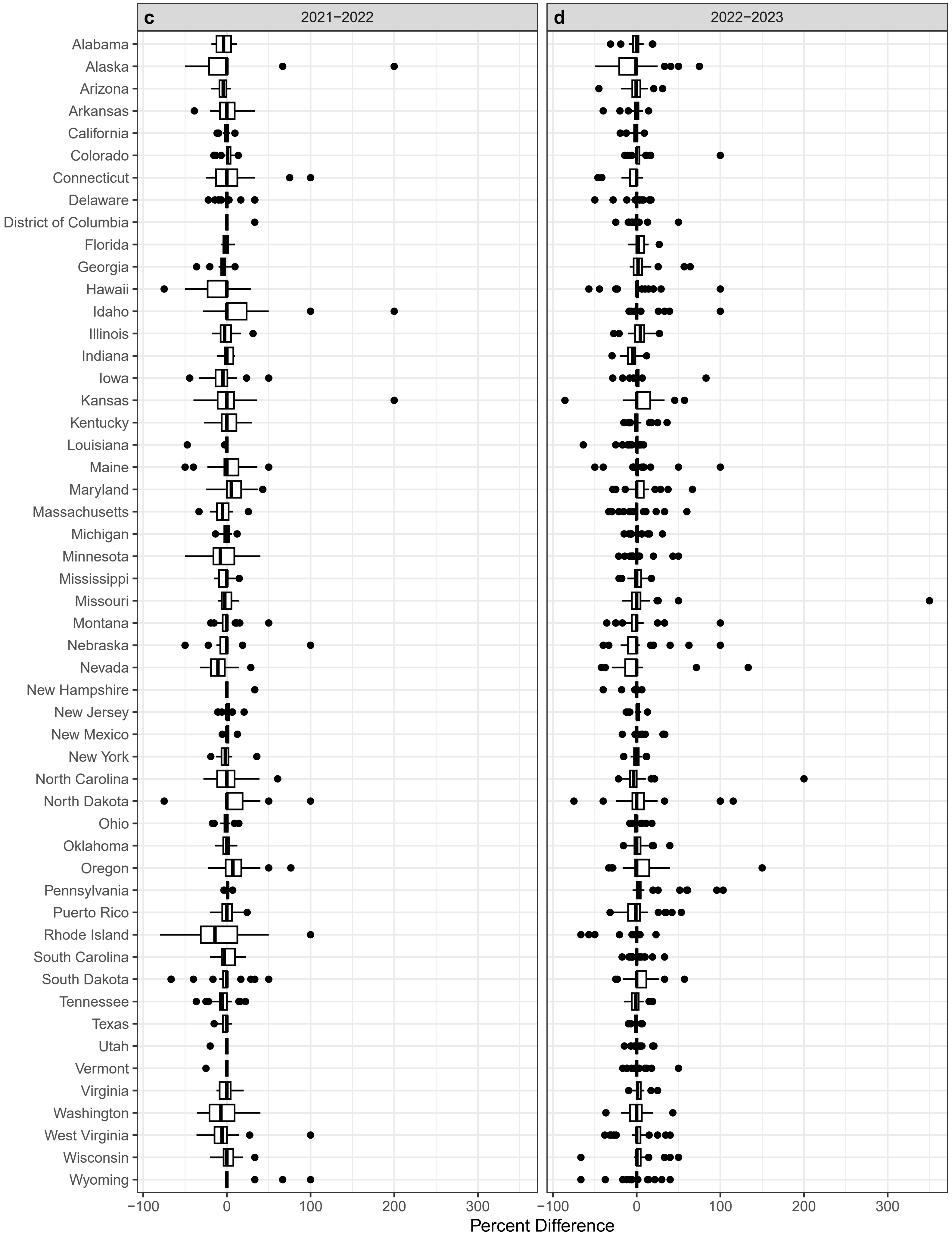
c
2021−2022
d
2022−2023
Alabama
Alaska
Arizona
Arkansas
California
Colorado
Connecticut
Delaware
District of Columbia
Florida
Georgia
Hawaii
Idaho
Illinois
Indiana
Iowa
Kansas
Kentucky
Louisiana
Maine
Maryland
Massachusetts
Michigan
Minnesota
Mississippi
Missouri
Montana
Nebraska
Nevada
New Hampshire
New Jersey
New Mexico
New York
North Carolina
North Dakota
Ohio
Oklahoma
Oregon
Pennsylvania
Puerto Rico
Rhode Island
South Carolina
South Dakota
Tennessee
Texas
Utah
Vermont
Virginia
Washington
West Virginia
Wisconsin
Wyoming
−100
0
100
200
300
Percent Difference

Supplementary Figure 4: Data revision analysis: U.S. weekly reported hospitalizations from the HHS−Protect system [3] with the initial date each weeks' data were pulled (vertical axis) and reported values as of subsequent dates (horizontal axis). Coloring for the number of hospitalizations was normalized on a common log scale. a) 2021−22 season, b) 2022−2023 season

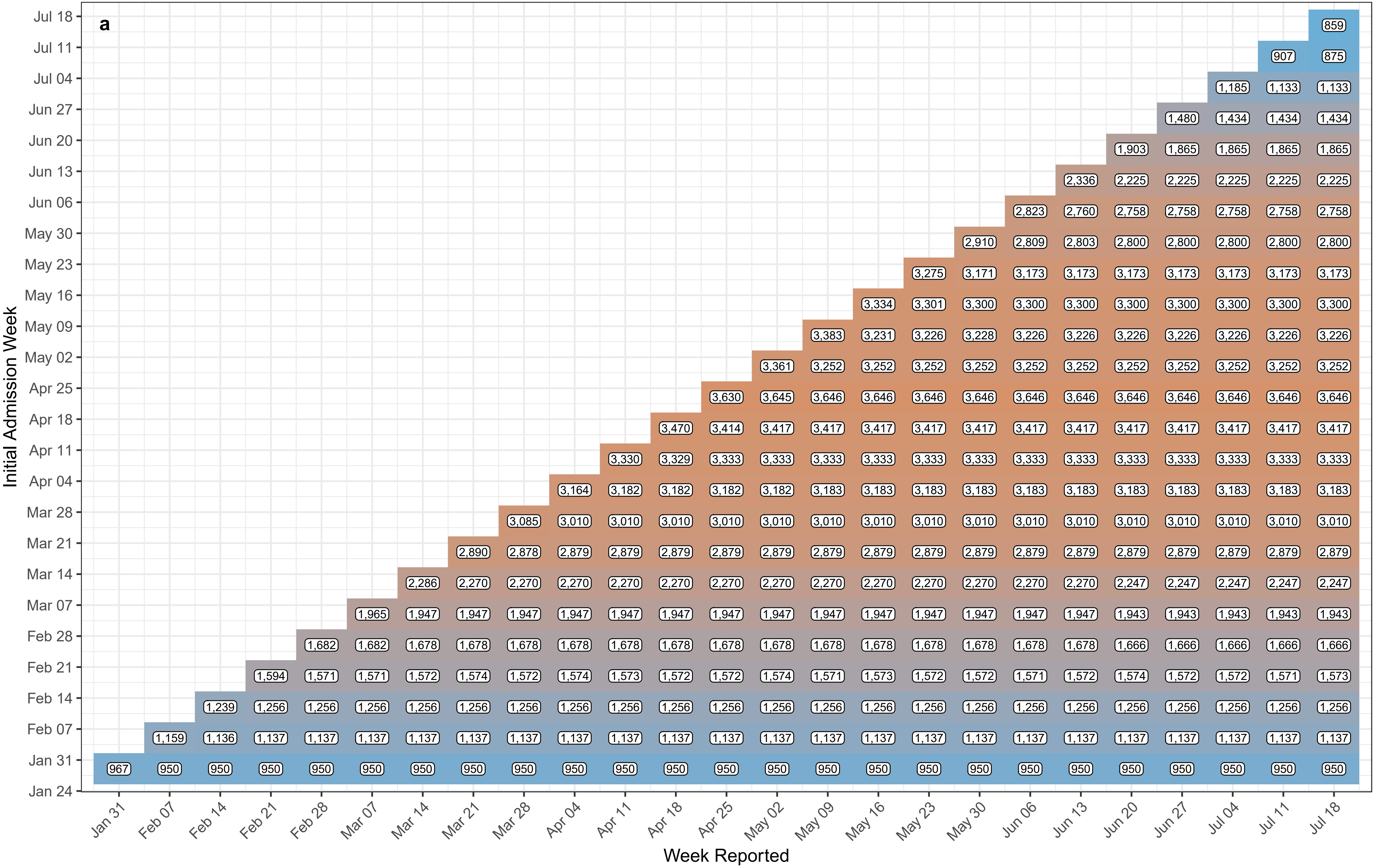

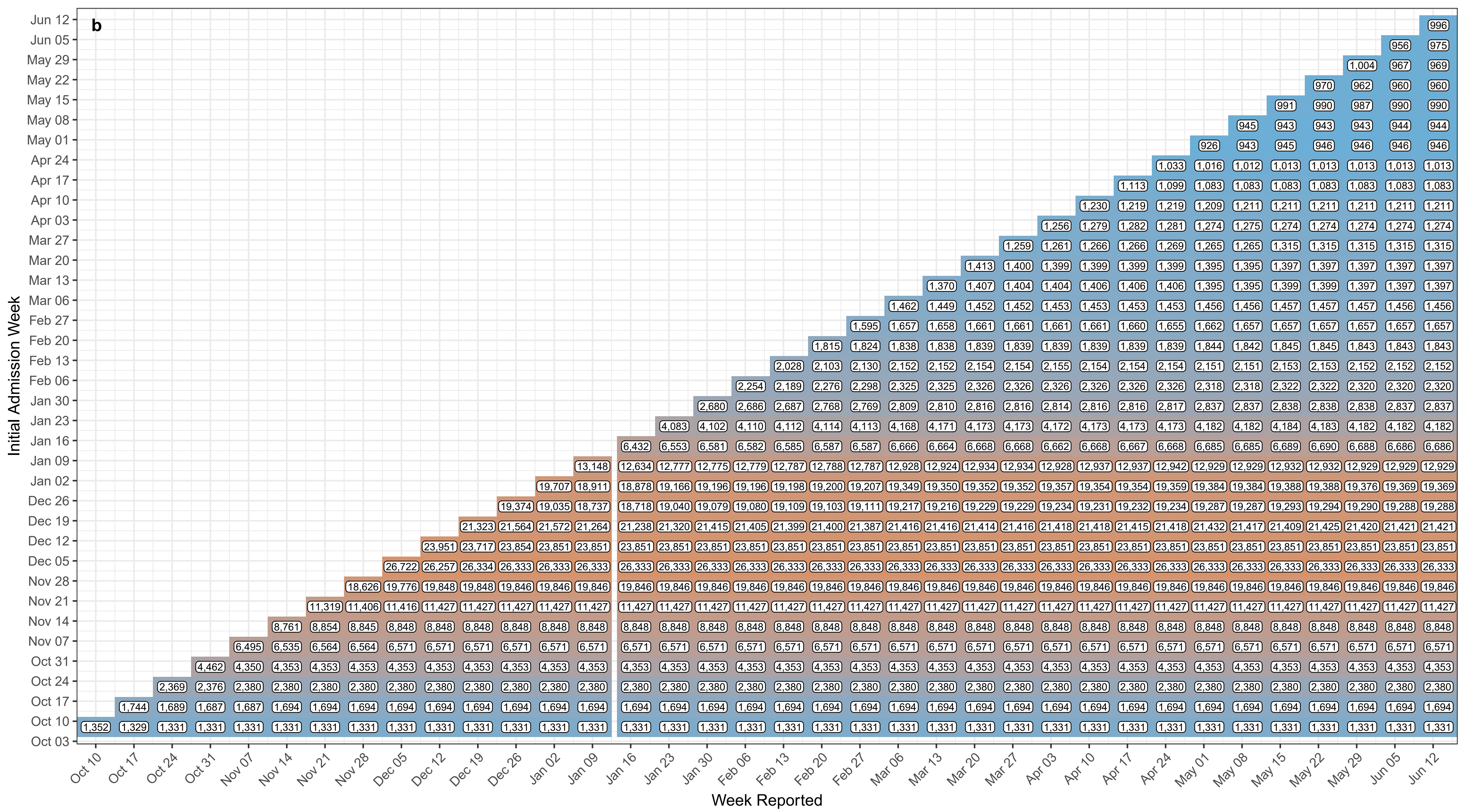
b
Initial Admission Week
Week Reported
Jun 12
Jun 05
May 29
May 22
May 15
May 08
May 01
Apr 24
Apr 17
Apr 10
Apr 03
Mar 27
Mar 20
Mar 13
Mar 06
Feb 27
Feb 20
Feb 13
Feb 06
Jan 30
Jan 23
Jan 16
Jan 09
Jan 02
Dec 26
Dec 19
Dec 12
Dec 05
Nov 28
Nov 21
Nov 14
Nov 07
Oct 31
Oct 24
Oct 17
Oct 10
Oct 03

Supplementary Analysis 2: Performance of log-transformed forecasts

Using the same methods described in the methods section, we analyzed performance based on log-transformed hospitalization counts ($\log(n+1)$). In terms of absolute and relative WIS, teams that outperformed the FluSight Ensemble in the regular analysis also outperformed the ensemble in the log-transformed analysis. For each season, the same five teams performed best in both analyses. In 2021-22, 8 models outperformed the baseline, compared to 6 when not transformed. In 2022-23, 11 models outperformed the baseline, compared to 12 when not transformed (Supplementary Table 3).

In terms of standardized rank, the FluSight Ensemble outperformed the other models for both seasons. Other models ranked similarly between analyses (Supplementary Figure 5).

Coverage was similar when using log-transformed hospitalization counts (Supplementary Table 4).

Supplementary Table 3: Performance metrics for teams meeting inclusion criteria

Model	Absolute WIS	Relative WIS	MAE	50% Coverage (%)	95% Coverage (%)	% of Forecasts Submitted
**2021-2022**						
CMU-TimeSeries	0.31	0.79	0.47	0.47	0.90	100.00
Flusight-ensemble	0.33	0.83	0.50	0.48	0.86	100.00
PSI-DICE	0.33	0.84	0.50	0.43	0.82	100.00
UMass-trends_ensemble	0.36	0.91	0.53	0.71	0.97	100.00
SGroup-RandomForest	0.38	0.97	0.59	0.47	0.95	100.00
CEID-Walk	0.39	0.98	0.55	0.52	0.82	88.89
CU-ensemble	0.39	0.98	0.57	0.44	0.77	100.00
GT-FluFNP	0.38	0.98	0.54	0.39	0.69	96.15
Flusight-baseline	0.40	1.00	0.56	0.49	0.83	100.00
SigSci-TSENS	0.40	1.01	0.55	0.38	0.72	95.73
IEM_Health-FluProject	0.40	1.02	0.58	0.50	0.85	100.00
LUcompUncertLab-TEVA	0.41	1.04	0.59	0.54	0.86	88.89
MOBS-GLEAM_FLUH	0.42	1.07	0.59	0.32	0.63	90.60
LUcompUncertLab-VAR2_plusCOVID	0.42	1.08	0.60	0.42	0.74	94.44
UVAFluX-Ensemble	0.45	1.14	0.57	0.38	0.64	98.50
UT_FluCast-Voltaire	0.45	1.15	0.71	0.50	0.91	99.25
LUcompUncertLab-VAR2K_plusCOVID	0.47	1.19	0.68	0.42	0.74	88.89
SGroup-SIkJalpha	0.49	1.24	0.63	0.18	0.46	100.00
LUcompUncertLab-VAR2	0.53	1.35	0.77	0.39	0.72	94.44
LUcompUncertLab-VAR2K	0.61	1.54	0.93	0.42	0.83	88.89
LosAlamos_NAU-CModel_Flu	0.63	1.62	0.90	0.26	0.59	100.00

Model	Absolute WIS	Relative WIS	MAE	50% Coverage (%)	95% Coverage (%)	% of Forecasts Submitted
GH-Flusight	0.74	1.88	0.79	0.06	0.13	94.44
SigSci-CREG	0.80	2.06	1.04	0.19	0.44	88.68
**2022-2023**						
MOBS-GLEAM_FLUH	0.37	0.66	0.54	0.38	0.75	92.90
CMU-TimeSeries	0.41	0.70	0.62	0.46	0.84	94.16
MIGHTE-Nsemble	0.41	0.70	0.58	0.48	0.79	95.99
PSI-DICE	0.42	0.71	0.60	0.45	0.77	100.00
Flusight-ensemble	0.44	0.74	0.62	0.51	0.77	100.00
JHU_IDD-CovidSP	0.49	0.82	0.69	0.47	0.78	82.54
UMass-trends_ensemble	0.49	0.83	0.69	0.60	0.87	100.00
CU-ensemble	0.51	0.85	0.69	0.45	0.66	84.62
SGroup-RandomForest	0.52	0.87	0.75	0.48	0.81	96.15
GT-FluFNP	0.53	0.90	0.66	0.52	0.72	94.82
UNC_IDD-InfluPaint	0.52	0.96	0.74	0.36	0.71	75.37
Flusight-baseline	0.59	1.00	0.73	0.41	0.68	100.00
UGA_flucast-OKeeffe	0.61	1.02	0.82	0.41	0.67	94.30
UVAFluX-Ensemble	0.61	1.02	0.75	0.20	0.39	98.08
VTSanghani-ExogModel	0.63	1.04	0.83	0.27	0.58	80.77
CEPH-Rtrend_fluH	0.58	1.07	0.89	0.38	0.74	88.46
SigSci-TSENS	0.66	1.11	0.88	0.50	0.68	92.97
SigSci-CREG	0.68	1.16	0.83	0.32	0.54	89.72
ISU_NiemiLab-Flu	0.71	1.25	0.93	0.29	0.51	76.92

Forecast targets are across all fifty states, D.C., and Puerto Rico for log-transformed forecast targets. The Absolute WIS column refers to the Weighted Interval Score for each model. All models with a relative WIS score less than one outperformed the baseline model when evaluated solely upon WIS. 95% and 50% coverage values are provided for the percent of times that reported weekly incidence values were within the 95% or 50% prediction intervals respectively, across all the forecast targets submitted by each team. The percent of forecasts submitted is determined by the number of forecast targets submitted by each team out of all possible forecast targets occurring within the duration of the evaluation period.

Supplementary Table 4: One-to-four-week coverage and one-to-four-week percent of coverage above 90% for teams meeting inclusion criteria.

Model	Relative WIS	% WIS Below Baseline	1 Wk Coverage	2 Wk Coverage	3 Wk Coverage	4 Wk Coverage	% Cov abv 90 (1 Wk)	% Cov abv 90 (2 Wk)	% Cov abv 90 (3 Wk)	% Cov abv 90 (4 Wk)
**2021-2022**										
CMU-TimeSeries	0.79	86.54	90.17	91.45	90.60	86.54	50.00	72.22	61.11	27.78
Flusight-ensemble	0.83	96.15	89.32	86.11	85.15	83.33	55.56	33.33	27.78	38.89
PSI-DICE	0.84	84.62	88.89	83.87	78.31	76.50	38.89	27.78	5.56	0.00
UMass-trends_ensemble	0.91	63.46	96.15	97.65	96.90	96.15	100.00	100.00	100.00	100.00
SGroup-RandomForest	0.97	57.69	95.41	94.87	94.66	94.12	88.89	88.89	83.33	88.89
CEID-Walk	0.98	61.54	82.09	83.77	81.01	81.85	37.50	37.50	31.25	37.50
CU-ensemble	0.98	57.69	79.59	80.66	76.50	71.90	16.67	11.11	0.00	0.00
GT-FluFNP	0.98	58.00	70.11	68.67	68.22	70.11	5.56	16.67	16.67	22.22
Flusight-baseline	1.00	0.00	82.26	84.19	82.48	81.62	27.78	22.22	22.22	22.22
SigSci-TSENS	1.01	52.00	74.11	73.44	70.54	69.20	11.11	5.56	5.56	5.56
IEM_Health-FluProject	1.02	48.08	91.45	86.54	82.59	78.21	72.22	38.89	22.22	22.22
LUcompUncertLab-TEVA	1.04	38.46	84.86	85.58	86.06	86.18	25.00	18.75	25.00	31.25
MOBS-GLEAM_FLUH	1.07	38.00	71.11	65.80	59.79	56.49	0.00	0.00	0.00	0.00
LUcompUncertLab-VAR2_plusCOVID	1.08	44.23	76.70	74.77	73.30	70.14	17.65	5.88	5.88	5.88
UVAFluX-Ensemble	1.14	15.38	66.05	65.51	62.58	60.95	11.11	0.00	0.00	0.00
UT_FluCast-Voltaire	1.15	26.92	94.73	90.96	89.13	90.42	83.33	72.22	55.56	61.11
LUcompUncertLab-VAR2K_plusCOVID	1.19	26.92	75.72	75.24	74.04	72.72	6.25	0.00	0.00	0.00
SGroup-SIkJalpha	1.24	7.69	40.28	45.73	48.08	48.29	0.00	0.00	0.00	0.00
LUcompUncertLab-VAR2	1.35	3.85	73.87	72.29	72.17	70.81	11.76	5.88	11.76	11.76
LUcompUncertLab-VAR2K	1.54	9.62	81.97	81.49	83.05	85.46	6.25	18.75	25.00	37.50
LosAlamos_NAU-CModel_Flu	1.62	11.54	65.28	59.29	56.52	54.06	5.56	0.00	0.00	0.00
GH-Flusight	1.88	3.85	18.33	12.90	11.99	10.63	0.00	0.00	0.00	0.00
SigSci-CREG	2.06	8.00	46.87	43.98	43.86	43.13	0.00	0.00	0.00	0.00

Model	Relative WIS	% WIS Below Baseline	1 Wk Coverage	2 Wk Coverage	3 Wk Coverage	4 Wk Coverage	% Cov abv 90 (1 Wk)	% Cov abv 90 (2 Wk)	% Cov abv 90 (3 Wk)	% Cov abv 90 (4 Wk)
**2022-2023**										
MOBS-GLEAM_FLUH	0.66	100.00	78.42	74.60	73.41	75.32	30.77	19.23	15.38	15.38
CMU-TimeSeries	0.70	92.31	84.13	84.84	84.76	84.13	50.00	57.69	65.38	65.38
MIGHTE-Nsemble	0.70	98.08	83.65	81.19	78.10	72.22	56.00	52.00	60.00	52.00
PSI-DICE	0.71	96.15	86.09	78.18	73.22	71.60	57.69	61.54	57.69	53.85
Flusight-ensemble	0.74	100.00	83.14	78.18	74.70	73.59	57.69	61.54	57.69	53.85
JHU_IDD-CovidSP	0.82	74.51	84.86	79.03	75.27	72.04	59.09	54.55	50.00	50.00
UMass-trends_ensemble	0.83	90.38	90.01	88.24	85.21	83.65	73.08	69.23	65.38	65.38
CU-ensemble	0.85	78.85	67.83	67.05	65.12	62.76	36.36	45.45	45.45	45.45
SGroup-RandomForest	0.87	90.38	88.08	81.38	78.23	76.46	68.00	64.00	64.00	60.00
GT-FluFNP	0.90	88.00	73.24	69.58	72.07	74.65	50.00	50.00	50.00	61.54
UNC_IDD-InfluPaint	0.96	76.47	71.93	70.56	71.05	71.84	55.00	40.00	55.00	50.00
Flusight-baseline	1.00	0.00	74.63	69.30	65.83	64.05	50.00	50.00	50.00	50.00
UGA_flucast-OKeeffe	1.02	50.98	76.31	67.69	62.75	60.39	40.00	36.00	28.00	28.00
UVAFluX-Ensemble	1.02	35.29	40.87	41.25	37.03	37.03	0.00	0.00	0.00	0.00
VTSanghani-ExogModel	1.04	32.69	63.00	58.15	55.31	55.13	0.00	0.00	0.00	4.76
CEPH-Rtrend_fluH	1.07	55.77	70.99	75.92	74.83	73.24	34.78	39.13	47.83	34.78
SigSci-TSENS	1.11	8.00	71.60	68.97	67.54	65.63	46.15	46.15	46.15	50.00
SigSci-CREG	1.16	20.00	61.83	54.74	50.70	48.15	38.46	38.46	34.62	34.62
ISU_NiemiLab-Flu	1.25	9.62	54.81	55.19	49.42	46.35	20.00	25.00	10.00	10.00

Supplementary Table 4: % WIS Below Baseline shows the percent of WIS values for each model below the corresponding FluSight-Baseline WIS.

The '% Cov abv 90' columns show the percent of weekly 95% coverage values that are greater than or equal to 90% for each model by horizon.

Supplementary Figure 5: Standardized rank of weighted interval score (WIS) for log-transformed hospital admission forecasts over all forecast jurisdictions and horizons (1- to 4-week ahead), for the FluSight ensemble and each team submitting at least 75% of the forecast targets, for season 2021-2022 (panel a), and 2022-2023 (panel b). See Supplementary Table 3 for qualifying teams and season metrics.

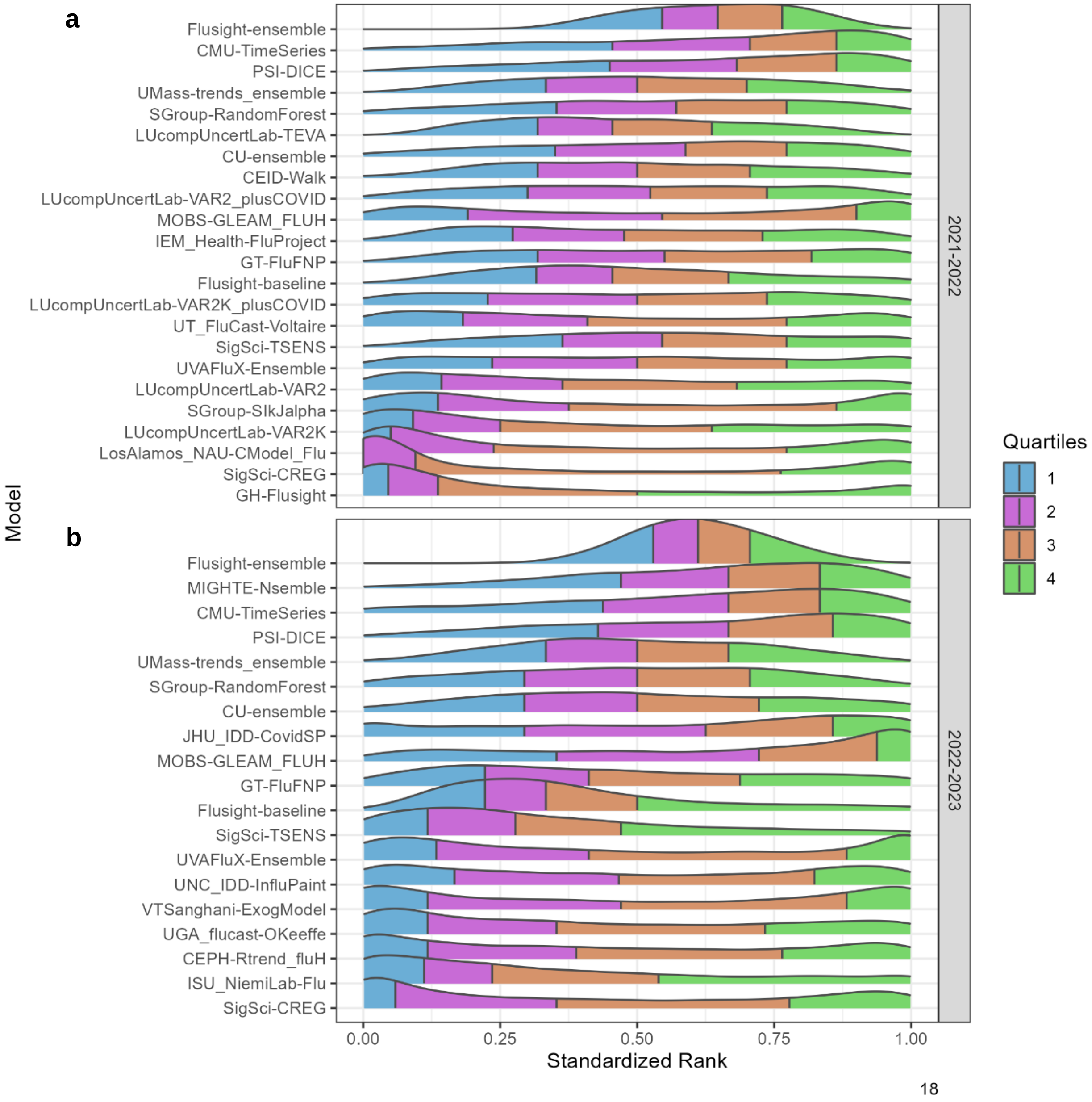

Supplementary Figure 6: Time series of absolute WIS of log-transformed forecasts. Note that the forecast evaluation period translates to 1-week ahead forecast target end dates from February 26 to June 25, 2022 (panel a), and October 22, 2022, to May 20, 2023 (panel b), and 4-week ahead forecast target end dates from March 19 to July 16, 2022 (panel c), and November 5, 2022, to June 10, 2023 (panel d). Weekly results for the FluSight baseline and ensemble models are shown in red and blue respectively. Results for individual contributing models are shown in light gray.

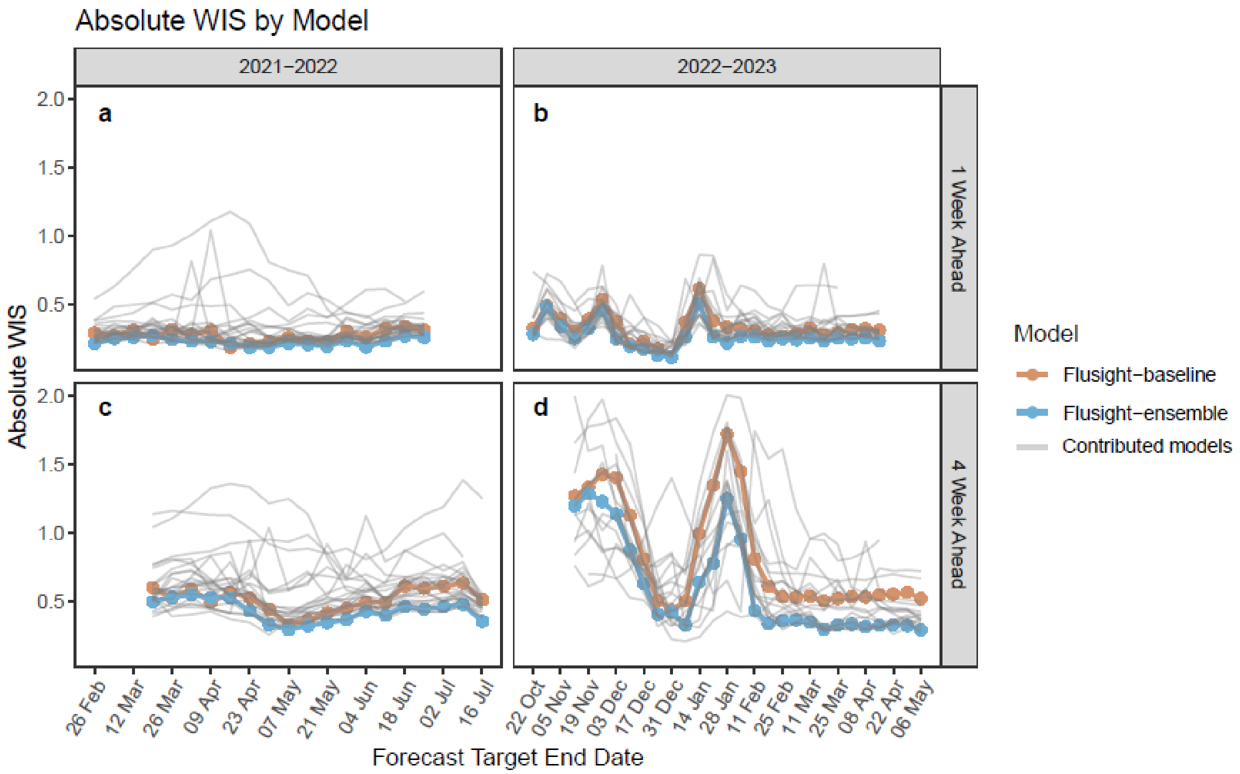

Supplementary Figure 7: State−level WIS values for each team relative to the FluSight baseline model using log−transformed hospitalization counts. The range of Relative WIS values below 1, in blue, indicate better performance than the FluSight baseline (white). Relative WIS values above 1, in red, indicate poor performance relative to the FluSight baseline. Teams are ordered on the horizontal axis from lowest to highest Relative WIS values for each season a) 2021-22 season, b) 2022-23 season.

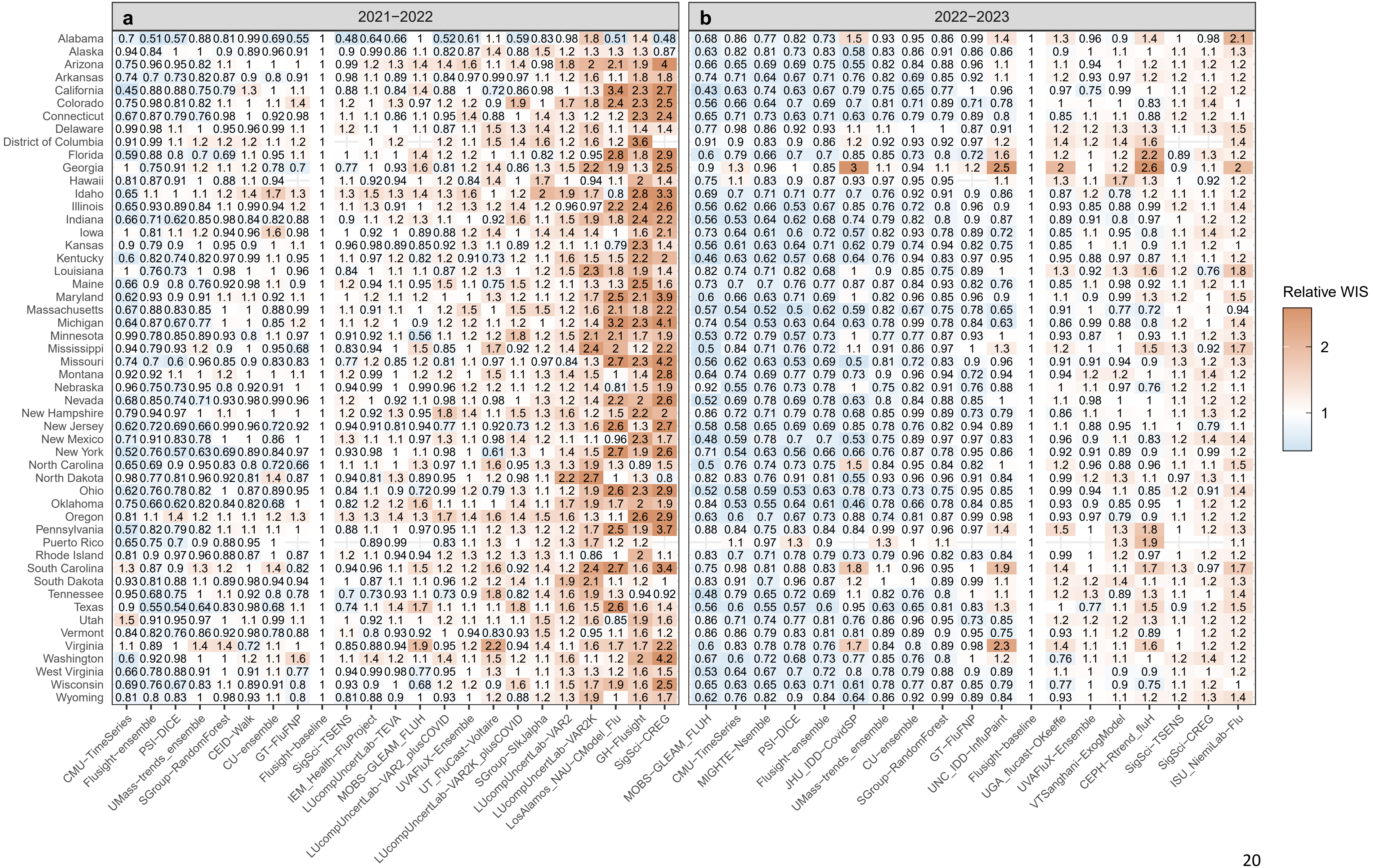

Supplement Analysis 3: Performance of national forecasts

When considering just national forecast targets, two additional teams met the eligibility criteria for inclusion in the end of season analysis. These models, as well as others submitting for a subset of jurisdictions, were included when generating ensembles during the season.

Supplementary Table 5: Performance metrics for teams submitting at least 75% of weekly national FluSight targets.

Model	Absolute WIS	Relative WIS	MAE	50% Coverage (%)	95% Coverage (%)	% of Forecasts Submitted
**2021-2022**						
PSI-DICE	296.70	0.71	421.38	55.56	88.89	100.00
UMass-trends_ensemble	301.67	0.72	485.97	45.83	84.72	100.00
CMU-TimeSeries	316.02	0.76	355.28	87.50	100.00	100.00
Flusight-ensemble	350.65	0.84	559.53	45.83	90.28	100.00
SigSci-TSENS	356.15	0.86	574.29	33.33	90.28	100.00
CU-ensemble	363.88	0.87	552.35	15.28	68.06	100.00
Flusight-baseline	418.70	1.00	684.62	26.39	87.50	100.00
UT_FluCast-Voltaire	440.00	1.07	633.96	37.50	84.72	100.00
GT-FluFNP	461.10	1.12	660.28	44.44	75.00	100.00
CEID-Walk	471.77	1.16	679.19	28.12	57.81	88.89
SGroup-RandomForest	495.62	1.20	727.54	69.44	100.00	100.00
MOBS-GLEAM_FLUH	486.49	1.22	727.21	17.65	63.24	94.44
IEM_Health-FluProject	613.93	1.48	785.61	41.67	97.22	100.00
LUcompUncertLab-TEVA	618.39	1.49	895.44	12.50	56.25	88.89
LUcompUncertLab-VAR2K	643.17	1.54	928.09	14.06	60.94	88.89
UVAFluX-Ensemble	757.94	1.78	655.74	70.83	94.44	100.00
LUcompUncertLab-VAR2K_plusCOVID	747.56	1.79	888.94	6.25	14.06	88.89
LUcompUncertLab-VAR2_plusCOVID	754.84	1.83	865.00	7.35	16.18	94.44
LUcompUncertLab-VAR2	863.37	2.10	1077.69	4.41	30.88	94.44
SGroup-SIkJalpha	1028.91	2.48	1543.18	15.28	48.61	100.00
GH-Flusight	1124.18	2.71	1141.29	0.00	1.47	94.44
LosAlamos_NAU-CModel_Flu	1395.41	3.43	1801.90	6.94	15.28	100.00
**2022-2023**						
MOBS-GLEAM_FLUH	1509.55	0.41	2270.89	42.31	90.38	100.00
CMU-TimeSeries	2043.33	0.55	3064.96	67.31	90.38	100.00
PSI-DICE	2087.92	0.57	2780.90	52.88	70.19	100.00
Flusight-ensemble	2466.05	0.67	3439.47	55.77	75.00	100.00
CU-ensemble	2800.38	0.68	3210.70	36.36	51.14	84.62
MIGHTE-Nsemble	2666.71	0.73	3480.24	53.00	68.00	96.15

SGroup-RandomForest	2869.94	0.78	3993.83	57.00	80.00	96.15
SigSci-TSENS	2891.23	0.78	3972.84	56.52	73.91	88.46
UMass-trends_ensemble	2914.47	0.78	3870.83	51.92	66.35	100.00
CEPH-Rtrend_fluH	2886.69	0.80	3208.62	33.70	57.61	88.46
UVAFluX-Ensemble	3007.81	0.80	4216.99	35.58	67.31	100.00
GT-FluFNP	2999.90	0.81	3516.60	57.69	68.27	100.00
UGA_flucast-OKeeffe	3466.83	0.94	4306.36	32.00	49.00	96.15
UNC_IDD-InfluPaint	3221.15	0.98	4092.70	36.25	75.00	76.92
Flusight-baseline	3724.31	1.00	4281.00	43.27	58.65	100.00
NIH-Flu_ARIMA	3910.19	1.01	4598.75	31.25	100.00	92.31
ISU_NiemiLab-Flu	5096.20	1.24	5870.45	17.50	37.50	76.92
JHU_IDD-CovidSP	8230.60	2.21	9494.40	31.82	63.64	84.62

The Absolute WIS column refers to the Weighted Interval Score for each model across all national forecast targets. All models with a relative WIS score less than one outperformed the baseline model when evaluated solely upon WIS. 95% and 50% coverage values are provided for the percent of times that reported weekly incidence values were within the 95% or 50% prediction intervals respectively, across all the forecast targets submitted by each team. The percent of forecasts submitted is determined by the number of forecast targets submitted by each team out of all possible forecast targets occurring within the duration of the evaluation period.

When only national forecast targets were considered, two additional models, PSI-DICE and UMass-trends_ensemble, outperformed the FluSight Ensemble in terms of relative WIS in the 2021-22 season. In contrast, one fewer models outperformed the FluSight Ensemble in terms of relative WIS in the 2022-23 season.

For national targets, the CMU-TimeSeries and PSI-DICE model forecasts outperformed the FluSight ensemble forecasts for both seasons in terms of relative WIS scores. Consistent differences in coverage values across models were not observed when comparing all jurisdiction results to national only results.

Supplementary References

1. Bosse N.I. et al, Evaluating Forecasts with scoringutils in R. arXiv. arXiv:2205.07090v1 (2022).
2. COVID-19 Reported Patient Impact and Hospital Capacity by State Timeseries (RAW). https://healthdata.gov/Hospital/COVID-19-Reported-Patient-Impact-and-Hospital-Capa/g62h-syeh. (2023).
3. COVID-19 Guidance for Hospital Reporting and FAQs For Hospitals, Hospital Laboratory, and Acute Care Facility Data Reporting, 11 June 2023, www.hhs.gov/sites/default/files/covid-19-faqs-hospitals-hospital-laboratory-acute-care-facility-data-reporting.pdf.

Disclaimers

Any use of trade, firm, or product names is for descriptive purposes only and does not imply endorsement by the U.S. Government. The findings and conclusions in this report are those of the authors and do not necessarily represent the views of the Centers for Disease Control and Prevention or the National Institutes of Health.
