# Supplementary material for: Evaluation of FluSight influenza forecasting in the 2021–22 and 2022–23 seasons with a new target laboratory-confirmed influenza hospitalizations
Source: Nat Commun. 2024 Jul 26;15:6289. doi: 10.1038/s41467-024-50601-9 (PMC11282251; doi:10.1038/s41467-024-50601-9)
Supplement: Supplementary file 3 — Reporting Summary [file 41467_2024_50601_MOESM3_ESM.pdf]

nature portfolio

Corresponding author(s): Sarabeth Mathis, Rebecca Borchering

Last updated by author(s): Jul 8, 2024

Reporting Summary

Nature Portfolio wishes to improve the reproducibility of the work that we publish. This form provides structure for consistency and transparency in reporting. For further information on Nature Portfolio policies, see our Editorial Policies and the Editorial Policy Checklist.

Statistics

For all statistical analyses, confirm that the following items are present in the figure legend, table legend, main text, or Methods section.

n/a	Confirmed	
☐	☒	The exact sample size (*n*) for each experimental group/condition, given as a discrete number and unit of measurement
☒	☐	A statement on whether measurements were taken from distinct samples or whether the same sample was measured repeatedly
☒	☐	The statistical test(s) used AND whether they are one- or two-sided<br>*Only common tests should be described solely by name; describe more complex techniques in the Methods section.*
☒	☐	A description of all covariates tested
☐	☒	A description of any assumptions or corrections, such as tests of normality and adjustment for multiple comparisons
☐	☒	A full description of the statistical parameters including central tendency (e.g. means) or other basic estimates (e.g. regression coefficient) AND variation (e.g. standard deviation) or associated estimates of uncertainty (e.g. confidence intervals)
☒	☐	For null hypothesis testing, the test statistic (e.g. *F*, *t*, *r*) with confidence intervals, effect sizes, degrees of freedom and *P* value noted<br>*Give P values as exact values whenever suitable.*
☒	☐	For Bayesian analysis, information on the choice of priors and Markov chain Monte Carlo settings
☒	☐	For hierarchical and complex designs, identification of the appropriate level for tests and full reporting of outcomes
☒	☐	Estimates of effect sizes (e.g. Cohen's *d*, Pearson's *r*), indicating how they were calculated

Our web collection on statistics for biologists contains articles on many of the points above.

Software and code

Policy information about availability of computer code

Data collection	Hospital admission target data were pulled via API in R the R language for statistical computing (version 4.0.3). Submitted forecast data were submitted and loaded through GitHub.
Data analysis	All analyses were conducted using the R language for statistical computing (version 4.0.3); submissions were scored using scoringutils (version 1.2.2) and corresponding code is available at the following GitHub repository (https://github.com/cdcepi/FluSight-manuscripts, 10.5281/zenodo.12625724).

For manuscripts utilizing custom algorithms or software that are central to the research but not yet described in published literature, software must be made available to editors and reviewers. We strongly encourage code deposition in a community repository (e.g. GitHub). See the Nature Portfolio guidelines for submitting code & software for further information.

Data

Policy information about availability of data

All manuscripts must include a data availability statement. This statement should provide the following information, where applicable:

- Accession codes, unique identifiers, or web links for publicly available datasets
- A description of any restrictions on data availability
- For clinical datasets or third party data, please ensure that the statement adheres to our policy

The forecasts from models used in this paper are available from the FluSight Forecast Hub GitHub repository (https://github.com/cdcepi/Flusight-forecast-data)

and the Zoltar forecast archive (https://zoltardata.com/project/299/viz). These are both publicly accessible. The target data are also available from HHS (https://healthdata.gov/Hospital/COVID-19-Reported-Patient-Impact-and-Hospital-Capa/g62h-syeh).

Research involving human participants, their data, or biological material

Policy information about studies with human participants or human data. See also policy information about sex, gender (identity/presentation), and sexual orientation and race, ethnicity and racism.

Reporting on sex and gender	n/a
Reporting on race, ethnicity, or other socially relevant groupings	n/a
Population characteristics	n/a
Recruitment	n/a
Ethics oversight	n/a

Note that full information on the approval of the study protocol must also be provided in the manuscript.

Field-specific reporting

Please select the one below that is the best fit for your research. If you are not sure, read the appropriate sections before making your selection.

☐ Life sciences ☐ Behavioural & social sciences ☒ Ecological, evolutionary & environmental sciences

For a reference copy of the document with all sections, see nature.com/documents/nr-reporting-summary-flat.pdf

Ecological, evolutionary & environmental sciences study design

All studies must disclose on these points even when the disclosure is negative.

Study description	This modeling study assesses the performance of laboratory-confirmed influenza hospital admission forecasts for the 2021-22 and 2022-23 United States influenza seasons of the FluSight forecast hub.
Research sample	FluSight is a collaborative hub that collected forecasts of laboratory-confirmed influenza hospital admissions from multiple, independent modeling teams. A baseline and ensemble model were also generated by the Centers for Disease Control (CDC) FluSight team. FluSight used an open call to solicit forecasts that met formatting guidelines from any group. Teams made forecasts at national and subnational (e.g. state-level) scales for 1 to 4 weeks ahead of observed laboratory-confirmed influenza hospital admission data. Forecasts included total number of estimated laboratory-confirmed influenza hospital admissions for each jurisdiction. Hospital admission data was downloaded from HHS (https://healthdata.gov/Hospital/COVID-19-Reported-Patient-Impact-and-Hospital-Capa/g62h-syeh) where daily counts of influenza hospital admissions were reported for each jurisdiction. These data were used as the target for forecasting and were available to forecasters.
Sampling strategy	This study assessed forecasts submitted to FluSight during the 2021-22 and 2022-23 influenza forecasting periods that met inclusion criteria of submitting at least 75% of targets.
Data collection	Target data were submitted to HHS by hospitals across the US. Data analyzed in this study are individual, ensemble, and baseline predictions from the FluSight Forecast Hub. Individual modeling teams submitted these forecasts via pull request to the public FluSight GitHub repository (https://github.com/cdcepi/Flusight-forecast-data). Ensemble and baseline forecasts generated by the CDC FluSight team are also included in this repository.
Timing and spatial scale	Forecasts of weekly influenza hospital admissions were openly solicited from existing COVID-19 and influenza forecasting networks every Monday from January 10, 2022, through June 20, 2022, for the 2021-22 season. For the 2022-23 season, forecasts were solicited every Monday from October 17, 2022, through January 9, 2023, then every Tuesday from January 17, 2023, through May 17, 2023. Forecasted jurisdictions included the U.S. national level, all fifty states, Washington D.C., and Puerto Rico.
Data exclusions	For inclusion in the primary analysis, forecasting teams must have submitted greater than or equal to 75% of the requested targets, for subnational jurisdictions, between the forecast evaluation period of February 21, 2022, to June 20, 2022 (total of 18 weeks) for 2021-22 or October 17, 2022, to May 15, 2023 (total of 30 weeks) for 2022-23. However the supplement includes model information for all submitting models and supplemental results for corresponding national-level forecasts. These results include additional teams which did not submit forecasts for the majority of states. Target data were reported daily by hospitals in each jurisdiction (all 50 states, Puerto Rico and Washington DC), aggregated by jurisdiction and published daily by HHS.
Reproducibility	This modeling study can be fully reproduced using data available at https://github.com/cdcepi/Flusight-forecast-data and code available at https://github.com/cdcepi/FluSight-manuscripts.
Randomization	This retrospective analysis is aimed to asses the performance of FluSight forecasts compared to observed data. FluSight accepted

Randomization	predictions from all groups willing to participate, and generated forecasts in real-time for situational awareness and public health preparedness. Therefore, randomization is not relevant.
Blinding	FluSight teams submit models individually so that a variety of perspectives and modeling approaches are represented. We have included information on the performance of the models to facilitate considerations of whether different classes of models perform similarly. Blinding was not relevant as the goal of this study was not to determine which types of model perform best, but rather if models are able to accurately predict hospital admissions across jurisdictions using a new forecasting target.

Did the study involve field work? ☐ Yes ☒ No

Reporting for specific materials, systems and methods

We require information from authors about some types of materials, experimental systems and methods used in many studies. Here, indicate whether each material, system or method listed is relevant to your study. If you are not sure if a list item applies to your research, read the appropriate section before selecting a response.

Materials & experimental systems

n/a	Involved in the study	
☒	☐	Antibodies
☒	☐	Eukaryotic cell lines
☒	☐	Palaeontology and archaeology
☒	☐	Animals and other organisms
☒	☐	Clinical data
☒	☐	Dual use research of concern
☒	☐	Plants

Methods

n/a	Involved in the study	
☒	☐	ChIP-seq
☒	☐	Flow cytometry
☒	☐	MRI-based neuroimaging

Plants

Seed stocks	n/a
Novel plant genotypes	n/a
Authentication	n/a
